# Challenges in the Diagnosis and Management of Patients with Fibrosing Interstitial Lung Disease

**DOI:** 10.1155/2022/9942432

**Published:** 2022-02-15

**Authors:** Leslie B. Tolle

**Affiliations:** Department of Pulmonary Medicine and Critical Care Medicine, Cleveland Clinic, Cleveland, OH, USA

## Abstract

Interstitial lung diseases (ILDs) are heterogeneous in their clinical presentation. Making a differential diagnosis of ILD requires a thorough medical history, clinical examination, serologies, high-resolution computed tomography (CT) scan, and, in some cases, bronchoalveolar lavage or surgical lung biopsy. Multidisciplinary discussion is recommended to improve diagnostic confidence. ILDs have a variable and unpredictable clinical course. Patients should be closely monitored to ensure that progression of ILD is detected promptly. This involves regular assessment of symptoms, lung function, and, where appropriate, high-resolution CT. Patients with some fibrosing ILDs may respond well to immunosuppressants, but even patients who respond well to immunosuppressants initially may later show deterioration despite appropriate management. The tyrosine kinase inhibitor nintedanib has been approved for the treatment of idiopathic pulmonary fibrosis, other chronic fibrosing ILDs with a progressive phenotype, and systemic sclerosis-associated ILD. The three case studies described in this article illustrate the challenges in the diagnosis and management of patients with fibrosing ILDs and the importance of taking a multidisciplinary and individualized approach to care, including regular monitoring and consideration of whether a patient's drug regimen needs to be changed when there is evidence of disease progression.

## 1. Introduction

Interstitial lung diseases (ILDs) are a large and heterogeneous group of disorders; some of which have a known cause (e.g., a connective tissue disease or exposure to an organic antigen) and some of which are idiopathic. Making a differential diagnosis of ILD requires a thorough medical history, clinical examination, serologies, high-resolution computed tomography (CT), and, in some cases, invasive testing such as bronchoalveolar lavage (BAL) or surgical lung biopsy [1–3]. Multidisciplinary discussion is recommended when the diagnosis is uncertain, such as when the clinical context and/or CT pattern are indeterminate, or biopsy results need to be integrated with clinical and CT features [1].

ILDs have a variable clinical course, which may involve periods of relative stability and periods of deterioration. Some ILDs may be responsive to immunosuppressant therapy, while for other ILDs, there is no evidence that immunosuppressant therapy is beneficial [4]. All patients with idiopathic pulmonary fibrosis (IPF) [2] and a proportion of patients with other ILDs [4] show a progressive fibrosing phenotype, characterized by worsening fibrotic abnormalities on CT, decline in lung function, worsening symptoms, and early mortality, despite conventional treatment. Regular monitoring of patients with ILDs in clinical practice is important so that patients with progressive lung fibrosis can be promptly identified and managed appropriately.

The three case studies described in this article illustrate the challenges in the diagnosis and management of patients with fibrosing ILDs and the importance of taking an individualized approach.

## 2. Case Reports

### 2.1. Case 1

A 61-year-old female lifetime nonsmoker with a remote history of uncomplicated and untreated ulcerative colitis was referred to our ILD clinic for undifferentiated ILD in April 2014. She had chronic dyspnea after developing an upper respiratory illness 8 years earlier. After initially responding well to inhaled corticosteroid therapy, her symptoms worsened and she underwent a CT scan, which suggested nonspecific inflammation, and a subsequent bronchoscopy. The results of the bronchoscopy were not available to us but were presumed to be nondiagnostic. The patient was empirically treated with a 6-month course of corticosteroids, and her symptoms resolved. She remained asymptomatic until 1 year prior to evaluation at our clinic, when she noted insidious onset of decreased exercise tolerance and had new chest X-ray findings and a decline in pulmonary function tests. She denied having cough, wheezing, or other cardiopulmonary symptoms and had no symptoms consistent with a connective tissue disease, gastroesophageal reflux disease, or dysphagia. Laboratory workup indicated negativity for antinuclear antibodies and rheumatoid factor. Her home was built in the 1880s and had visible mold at the time of her initial presentation but had since been remediated. She denied any other exposures to chemicals, inhalants, or dusts. She had not noticed any change in symptoms when out of her usual environment. Her physical exam was unremarkable. At evaluation at our clinic in April 2014, the patient's forced vital capacity (FVC) was within the normal range, but had declined since it was measured at her local clinic in 2007 (Table 1).

The report of the patient's first CT scan in 2006 noted florid, bilateral, somewhat patchy, ground-glass opacities with slight basal predominance (images unavailable). No CT scans taken between 2006 and referral to our clinic in 2014 were available. An HRCT taken at our clinic showed upper lobe predominant peribronchovascular and peripheral fibrosis with some mild ground-glass opacities and mosaicism, as well as air trapping on expiratory images. Lung bases were spared. There was no significant lymphadenopathy. No honeycombing was seen. The patient was referred for bronchoscopy with BAL and transbronchial biopsies. BAL infectious studies were negative. No cell count or differential was available. Transbronchial biopsy showed a chronic interstitial inflammatory infiltrate composed predominantly of small lymphocytes and plasma cells, poorly formed nonnecrotizing granulomas, and occasional isolated multinucleated giant cells. Special stains for mycobacteria and fungi were negative.

**Figure 1:**
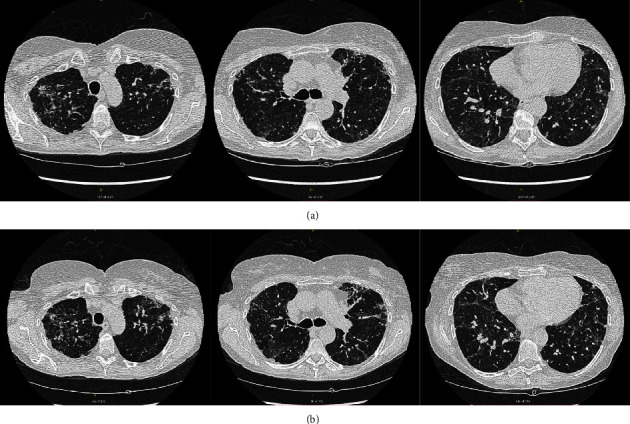
High-resolution computed tomography scans in a patient diagnosed with fibrotic hypersensitivity pneumonitis (Case 1) showing progression of fibrosis between (a) 2018 and (b) 2019 despite treatment with corticosteroids and mycophenolate sodium.

Based on these findings, the patient was given a diagnosis of fibrotic hypersensitivity pneumonitis due to an unknown antigen, possibly the mold in her home that had since been remediated. She performed a thorough assessment of her environment, and no other exposure was identified. She was treated with a prolonged course of corticosteroids (prednisone 40 mg daily, which was weaned off over the course of 6 months) with resolution of her symptoms, but four years later, she returned to our clinic with recurrence of her symptoms and a dramatic fall in lung function tests. Prednisone was restarted, with the addition of steroid-sparing immunosuppression with mycophenolate sodium, but she continued to progress on spirometry (Table 1) and on imaging (Figure 1). After more than two years on mycophenolate sodium, nintedanib has recently been added to her treatment regimen.

### 2.2. Case 2

A 66-year-old female minimal former smoker presented to our ILD clinic in June 2016 with biapical pulmonary parenchymal fibrotic changes with ground-glass opacities on CT, dyspnea, and a productive cough. Her symptoms had started after an upper respiratory tract infection 3 months prior. She had a mMRC (Modified Medical Research Council) dyspnea score of 1. She had no other cardiopulmonary or infectious symptoms, nor any symptoms suggestive of connective tissue disease. She had no occupational or environmental exposures, nor had she received pulmonary-toxic medications. Her exam was largely unremarkable other than a retracted suprasternal notch. Autoimmune serologies were negative. Spirometry suggested mild restriction with mild diffusion impairment, consistent with her parenchymal lung disease (Table 2).

**Figure 2:**
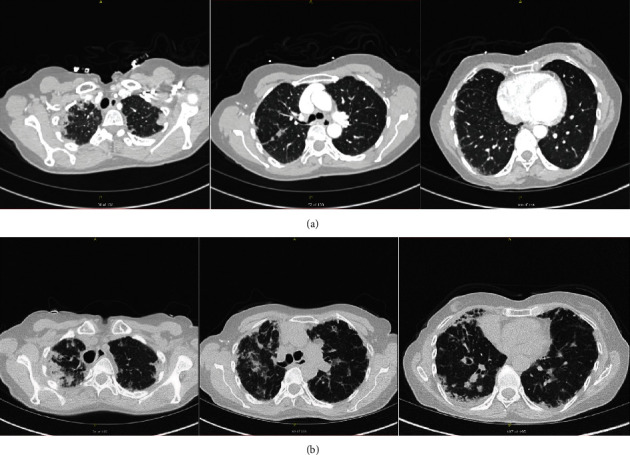
High-resolution computed tomography scans in a patient diagnosed with pleuroparenchymal fibroelastosis (Case 2). (a) Scans in 2015 showed interstitial changes bilaterally, most pronounced in the lung apices, with soft tissue thickening along the major fissure of the right lung between the middle and upper lobes and mild consolidation of the right lung apex. (b) Scans in 2020 showed progression of fibrosis.

Her initial CT in 2015 showed interstitial changes bilaterally, most pronounced in the lung apices (Figure 2(a)). There was soft tissue thickening along the major fissure of the right lung between the middle and upper lobes and mild consolidation of the right lung apex. There was no mediastinal or hilar lymphadenopathy or pleural effusion. A CT scan 6 months later showed similar findings that had mildly progressed. The patient was referred for bronchoscopy with BAL and transbronchial biopsy. BAL grew *Haemophilus influenzae*, prompting a course of amoxicillin-clavulanate, after which she was left with a minimal dry cough. Transbronchial biopsy showed minimal focal chronic inflammation and fibrosis, which was thought to be nondiagnostic.

Her case was discussed at a multidisciplinary ILD conference where she was given a consensus diagnosis of idiopathic pleuroparenchymal fibroelastosis (PPFE). Known causes of PPFE were historically absent or ruled out. Based on the characteristic nature of her CT scan, a surgical lung biopsy was not recommended. The group recommended initiating antifibrotic therapy, but she declined based on her preserved functional status and the side effect profile of antifibrotic medications. Over time, her disease slowly progressed on spirometry (Table 2) and on imaging (Figure 2(b)). She is now considering starting antifibrotic therapy and has been referred for evaluation for lung transplantation.

### 2.3. Case 3

A 60-year-old male former smoker was referred to our ILD clinic for undiagnosed ILD in October 2015. He had developed acute onset of dyspnea on exertion 4 months prior to evaluation at our clinic. He was admitted to the hospital locally, where he had a bronchoscopy that did not show infection. He was then treated with prednisone (information on dose and course unavailable). His symptoms improved and he was referred for follow-up. On further questioning, he denied having rash, mouth sores, digital ulcers, sun sensitivity, myalgias, Raynaud's phenomenon, or alopecia. He complained of bilateral chronic knee pain but did not have erythema, warmth, or swelling. He had not been on any pulmonary-toxic medications and denied any inhalational exposures. He worked as a cook in a Chinese restaurant. His physical exam was only significant for bibasilar rales and early clubbing. Spirometry suggested moderate restriction and mild diffusion impairment (Table 3).

**Figure 3:**
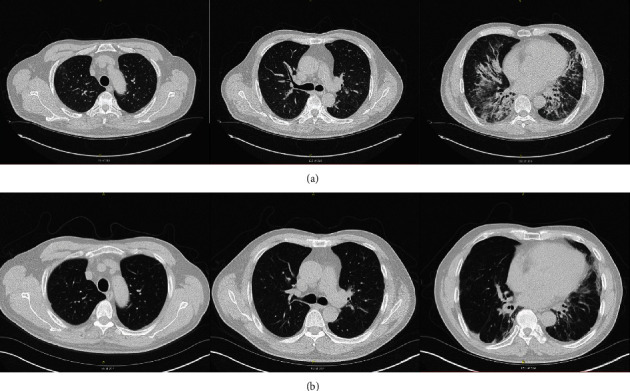
High-resolution computed tomography scans in a patient diagnosed with antisynthetase syndrome (Case 3). (a) Scans in 2015 showed basilar predominant bronchiectatic changes with a significant amount of peribronchial ground-glass opacity, as well as some coarse subpleural reticular opacities, consistent with a nonspecific interstitial pneumonia (NSIP) or organizing pneumonia (OP) pattern. (b) Scans in 2019 showed improvement following treatment with mycophenolate mofetil.

The initial HRCT showed basilar predominant bronchiectatic changes with a significant amount of peribronchial ground-glass opacity, as well as some coarse subpleural reticular opacities (Figure 3(a)). Middle lobe and lingula demonstrated some peripheral sparing. Overall, this CT was thought to be most consistent with a nonspecific interstitial pneumonia (NSIP) or organizing pneumonia (OP) pattern. His workup included autoimmune serologies, which were positive only for anti-Jo 1 antibody (1.6 antibody index (AI), upper limit of normal: <1.0).

Based on his CT pattern showing NSIP/OP and positivity for anti-Jo 1 antibody, the patient was given a diagnosis of antisynthetase syndrome. A surgical lung biopsy was discussed but the patient declined. He received prednisone for 3 months, with marked improvement in symptoms but no change in spirometry (Table 3). After prednisone was discontinued, his symptoms worsened and his diffusing capacity for carbon monoxide (DL_CO_) declined. Echocardiogram showed no evidence of pulmonary hypertension. Prednisone was restarted, and mycophenolate mofetil was initiated as steroid-sparing therapy. His symptoms improved and DL_CO_ rebounded (Table 3). Over time, prednisone was tapered off and he has been maintained on mycophenolate mofetil. His symptoms remain minimal and spirometry continues to improve slowly (Table 3). A repeat HRCT scan (Figure 3(b)) showed some improvement compared with the initial scan taken more than 4 years earlier, consistent with his clinical course.

## 3. Discussion

ILDs are heterogeneous in their clinical presentation and have an unpredictable clinical course. The three case reports described in this article highlight the challenges in the diagnosis and management of fibrosing ILDs and the importance of regular monitoring so that treatment can be changed or escalated in patients whose ILD is progressing.

A diagnosis of ILD should be made based on careful evaluation of a high-resolution CT scan, clinical assessment, and serologic tests [1–3]. In the appropriate clinical context, a diagnosis can be made without a biopsy being performed [1, 2]. For example, in Case 2, a diagnosis of PPFE was made based on the characteristic nature of the patient's CT. In Case 3, the patient had a pattern on high-resolution CT consistent with NSIP/OP, the most common patterns observed in patients with antisynthetase syndrome [5, 6], in addition to positivity for anti-Jo 1 antibody, enabling a diagnosis of antisynthetase syndrome to be made in the absence of histopathological data. In Case 1, the findings of a transbronchial biopsy were valuable in reaching a diagnosis of hypersensitivity pneumonitis when a culprit exposure was unclear. The patient's imaging and subsequent clinical course showed that she had progressive fibrotic disease.

Making a differential diagnosis of ILD can be challenging, particularly in the case of rarer ILDs such as PPFE, as diagnosed in Case 2 [7, 8]. Multidisciplinary discussion is recommended in cases where clinical, radiological, serological, and, where available, histopathological data do not clearly lead to a specific diagnosis and can help to improve diagnostic confidence [1–3, 9]. However, it has been estimated that 10-20% of cases of ILD remain unclassifiable even after multidisciplinary discussion [10]. In these cases, a “working diagnosis” may be made to guide management, but the evidence supporting different diagnoses should be revisited regularly [1]. The longitudinal behavior of a patient's ILD may help to inform a more specific diagnosis.

Patients with some fibrosing ILDs may respond well to immunosuppressants, at least initially. However, as demonstrated by the cases described in this article, patients who respond well to immunosuppressant therapy in the short term may later show deterioration, despite appropriate management. Close monitoring of patients based on regular assessment of lung function, symptoms, and, where appropriate, high-resolution CT is important to identify patients whose ILD is progressing. Patients with a progressive fibrosing phenotype of ILD are at high risk of further progression and mortality [11–14]. Even for connective tissue disease-related ILDs, the evidence that continued immunosuppression slows the progression of fibrosing ILDs is limited. Data from randomized double-blind controlled trials are only available for patients with systemic sclerosis-associated ILD [15–18]. Immunosuppression has not been shown to slow the progression of fibrotic hypersensitivity pneumonitis [19, 20] and should not be used for chronic treatment of IPF [21]. The tyrosine kinase inhibitor nintedanib has been approved by the FDA for the treatment of IPF, other chronic fibrosing ILDs with a progressive phenotype (irrespective of diagnosis), and systemic sclerosis-associated ILD, based on the results of randomized placebo-controlled trials showing that it approximately halved the rate of decline in FVC over 52 weeks in these patients [22–25]. Pirfenidone has been approved for the treatment of IPF, also based on reducing decline in FVC over 52 weeks [26]. While antifibrotic therapy is now widely regarded as standard of care for IPF, given the inexorably progressive nature of IPF and its poor prognosis, there is no established treatment algorithm for other fibrosing ILDs. Decisions on when to initiate or escalate therapy should be made considering the severity of ILD and evidence of progression, risk factors for progression, other manifestations of disease, and the patient's preferences. In patients with ILDs related to connective tissue disease, it is important to consider all disease manifestations when making decisions about management, with input from specialists in both pulmonology and rheumatology [27]. Patients with fibrosing ILDs benefit from a holistic approach to management, which may include nonpharmacological therapies such as pulmonary rehabilitation [28], supplemental oxygen [29], and vaccinations or enrollment in clinical trials. Supportive/palliative care should be provided as appropriate throughout the course of the disease [30]. Lung transplantation should be evaluated for patients with IPF or with other ILDs that have not responded to treatment at an early stage [31].

In conclusion, as shown by the case reports described in this article, the differential diagnosis and management of fibrosing ILDs present challenges to clinicians, which can most effectively be addressed by taking a multidisciplinary and individualized approach.

## Figures and Tables

**Table 1:** Pulmonary function tests from Case 1.

	8 May 2007	31 March 2014	11 July 2014	22 October 2014	1 November 2018	11 October 2019	15 October 2020
FVC							
L	3.26	2.73	3.04	2.95	2.41	2.04	1.78
% predicted	97	80	89	92	75	53	n/a
FEV_1_							
L	2.30	2.02	2.11	1.88	1.83	n/a	n/a
% predicted	84	77	80	72	71	n/a	n/a
FEV_1_/FVC	71	74	69	64	76	n/a	n/a
DL_CO_							
mL/mmHg/min	13.3	14.14	14.60	13.5	13.62	11.2	10.3
% predicted	63	64	67	80	85	77	n/a

DL_CO_: diffusing capacity for carbon monoxide; FEV_1_: forced expiratory volume in one second; FVC: forced vital capacity; n/a: not available.

**Table 2:** Pulmonary function tests from Case 2.

	2 June 2016	27 April 2017	26 April 2018	23 May 2019	1 November 2019	23 July 2020
FVC						
L	2.32	2.36	2.40	2.21	2.11	1.80
% predicted	70	72	74	74	71	61
FEV_1_						
L	1.68	1.71	1.67	1.68	1.61	1.54
% predicted	67	69	68	72	69	67
FEV_1_/FVC	73	73	70	76	76	85
DL_CO_						
mL/mmHg/min	14.93	17.13	14.57	13.76	14.86	11.50
% predicted	69	80	69	65	71	55

DL_CO_: diffusing capacity for carbon monoxide; FEV_1_: forced expiratory volume in one second; FVC: forced vital capacity.

**Table 3:** Pulmonary function tests from Case 3.

	20 October 2015	3 March 2016	23 June 2016	9 December 2016	9 June 2017	19 July 2018	10 January 2019	19 December 2019	23 July 2020
FVC									
L	2.56	2.40	2.32	2.46	2.60	2.67	2.67	2.64	2.63
% predicted	52	51	49	52	55	57	58	67	66
FEV_1_									
L	2.01	1.94	1.80	1.94	1.99	1.99	2.06	1.94	2.11
% predicted	54	54	50	55	57	57	59	62	67
FEV_1_/FVC	79	81	77	79	77	75	77	74	80
DL_CO_									
mL/mmHg/min	17.86	17.29	14.41	20.34	18.11	22.07	22.25	21.64	25.00
% predicted	62	61	51	72	65	80	80	79	97

DL_CO_: diffusing capacity for carbon monoxide; FEV_1_: forced expiratory volume in one second; FVC: forced vital capacity.

## Data Availability

Additional anonymized data on these case studies may be available on request. Please contact the author.
